# Mast Cells and Resistance to Immunotherapy in Cancer

**DOI:** 10.1007/s00005-023-00676-x

**Published:** 2023-04-11

**Authors:** Domenico Ribatti

**Affiliations:** Department of Translational Biomedicine and Neuroscience, University of Bari Medical School, Piazza Giulio Cesare, 11, 70125 Bari, Italy

**Keywords:** Immunotherapy, Mast cells, Resistance, Tumor growth

## Abstract

Mast cells are involved in tumor growth and their mediators exert both pro- and anti-tumorigenic roles in different human cancers. The identification of defined immunosuppressive pathways that are present in the tumor microenvironment has pointed therapeutic strategies that may promote inflammation and/or innate immune activation in this context. Mast cells can contribute to the immune suppressive tumor microenvironment and may also enhance anti-tumor responses. This review article is focused on the analysis of the mechanisms of the role of mast cells in resistance to immunotherapy in cancer.

## Introduction

Tumor microenvironment shows a diversity of tumor cells, stromal cells, and several types of immune cells. Tumor microenvironment can inhibit tumor growth and suppress or revert the malignant phenotype. In the meantime, signals from the microenvironment can lead to initiation and promotion of neoplastic transformation of normal cells. The identification of defined immunosuppressive pathways that are present in the tumor microenvironment has pointed therapeutic strategies that may promote inflammation and/or innate immune activation in this context.

**Fig. 1:**
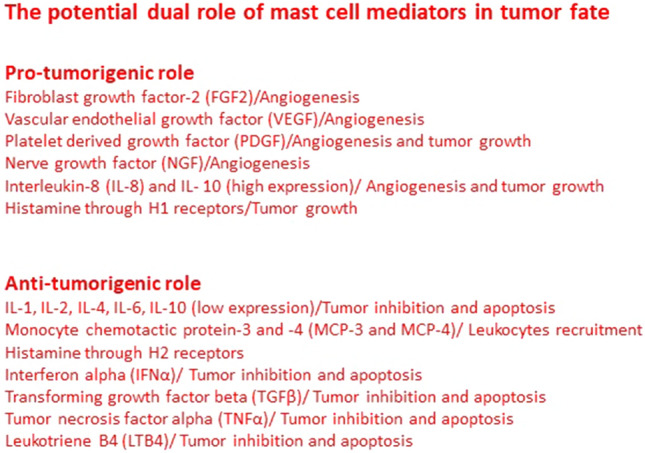
The potential dual role of mast cell mediators in tumor fate

Among the inflammatory cells present in tumor microenvironment, mast cells and their mediators are majorly involved in pro-tumorigenic roles, however, they also exert anti-tumorigenic roles (Ribatti 2022) (Fig. 1). Mast cells promote tumor growth by releasing cytokines and growth factors, such as fibroblast growth factor (FGF)-2, vascular endothelial growth factor (VEGF), platelet-derived growth factor (PDGF), nerve growth factor (NGF), interleukin (IL)-8, and IL-10 (high expression), histamine through H1 receptors, tryptase and chymase. In contrast, mast cells inhibit tumor growth, by releasing IL-1, IL-2, IL-4, IL-6, IL-10 (low expression), monocyte chemotactic protein-3 and -4 (MCP-3 and -4), histamine through H2 receptors, interferon alpha  (IFNα), transforming growth factor (TGF)-β, tumor necrosis factor (TNF)-α, and leukotriene B4 (Ribatti and Crivellato 2009) (Fig. 1).

Mast cells are recruited in the tumor microenvironment by stem cell factor (SCF) secreted by tumor cells and release different angiogenic factors and proteases involved in the promotion of tumor angiogenesis and invasiveness (Ribatti and Crivellato 2009). Mast cells produce several pro-angiogenic factors, including FGF-2, VEGF, IL-8, TNF-α, TGF-β, and NGF, and migrate in vivo and in vitro in response to VEGF and placental growth factor-1 (PlGF-1) (Ribatti and Crivellato 2009). Mast cells and their granules can stimulate an intense angiogenic response in vivo, partly inhibited by anti-FGF-2 and -VEGF antibodies (Ribatti et al. 2001). Among the proteases secreted by mast cells, tryptase stimulates the proliferation of endothelial cells and vascular tube formation in vitro, degrades connective tissue matrix, and activates matrix metalloproteinases (MMPs) and plasminogen activator, which in turn favor the release of VEGF and FGF-2 from their matrix-bound state (Blair et al. 1997). Moreover, the expression of mast cell chymase and tryptase correlated with angiogenesis during tumor progression (de Souza et al. 2012).

Mast cells express high levels of the tyrosine kinase receptor cKIT, and SCF, the ligand for cKIT, is involved in mast cell development and growth (Ribatti and Crivellato 2014). Moreover, SCF enhances tumor growth through and increased release of VEGF, IL-10, and TNF-α (Huang et al. 2008).

## Inhibition of Mast Cells as a Therapeutic Strategy in Tumor Treatment

Therapeutic approaches used in diseases in which mast cell numbers are increased include tyrosine kinase inhibitors (midostaurin, nilotinib, imatinib, sunitinib, sofrafenib, and dasatinib) to target the cKIT tyrosine kinase receptor action and mast cell tryptase inhibitors (gabexate mesylate, nafamostat mesylate, and tranilast) (Wishart et al. 2018). The first tyrosine kinase inhibitor introduced into the clinic, STI571 (imatinib mesylate, Gleevec), has inhibitory activity against the signaling cascade activated by the KIT receptor (CD117) (Heinrich et al. 2000). Imatinib has been used against gastrointestinal stromal tumors and against metastatic melanoma with cKIT mutations (Hodi et al. 2013; Kitamura and Hirotab 2004).

Masatinib is a tyrosine kinase inhibitor targeting cKIT receptor, clinically developed and approved for treatment of recurrent or unresectable grade III dog mast cell tumors (Dubreuil et al. 2009). A humanized anti-cKIT monoclonal antibody decreased mast cell degranulation and reduced mast cell number in canine mast cell tumors (London et al. 2017). Mast cell-stabilizing agents, such as cromolyn sodium, have been used in different pre-clinical models of solid tumors. In a xenograft mouse model of thyroid cancer, treatment with cromolyn reduced tumor growth (Melillo et al. 2010).

## Cancer Immunotherapy

Immunotherapy treatments are designed to target the immune system itself by triggering an effective immune attack against tumor cells. Cancer immunotherapies are classified as active and passive treatments. Active treatments include vaccines designed to induce tumor cell recognition. Passive treatments, on the other hand, imply direct administration of antibodies and T cells. In this context, immune checkpoint inhibitors and adoptive T-cell therapy are among the most innovative approaches.

Under normal physiologic conditions, immune checkpoints function as negative feedback to regulate inflammatory responses following T-cell activation to prevent autoimmunity and limit immune responses following antigen stimulation. The major immune check point classes are cytotoxic T lymphocyte antigen-4  (CTLA-4), programmed death (PD)-1, and programmed death-ligand (PDL)-1. The therapeutic use of antibodies that neutralize CTLA-4, PD-1, or PDL-1 binding sustain T-cell activation in the tumor microenvironment and improve cytotoxic response and protection against tumor growth. Since the first approval of the immune checkpoint inhibitor ipilimumab for melanoma, which targets the anti CTLA-4 checkpoint, different immune checkpoint inhibitors that target a PD-1 or PDL-1 for a wide range of cancer indications has been described (Robert 2020). The immune checkpoint inhibitors have provided significant clinical benefit including improvement in overall survival for some of the most aggressive cancers, including melanoma, non-small-cell lung cancer and renal cancer (Siu et al. 2017); however, the overall objective response rate gained by immune checkpoint inhibitors as a monotherapy remains suboptimal, ranging between 20 and 30%, and overall survival and toxicity profile need to be improved (Martins et al. 2019; Nishino et al. 2017).

**Fig. 2:**
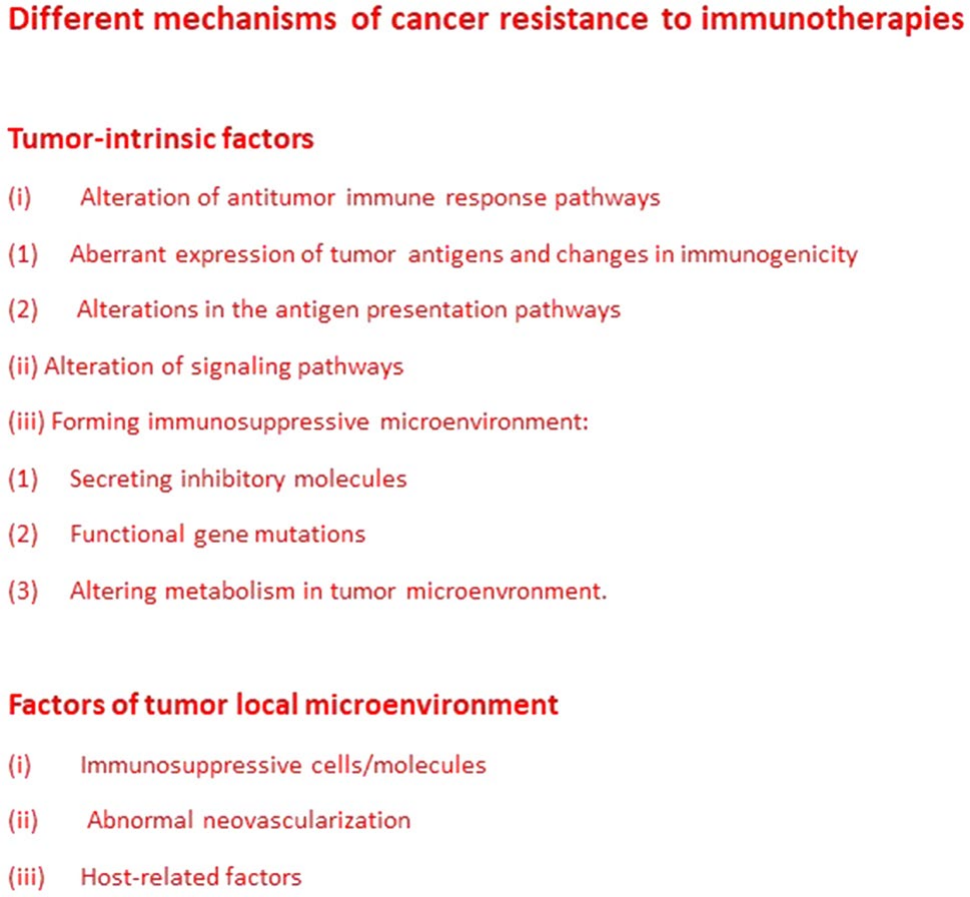
Different mechanisms of cancer resistance to immunotherapies

The mechanisms of cancer resistance to different immunotherapies are very complex and are summarized in Fig. 2.

## Mast Cells and Resistance to Immunotherapy in Cancer

Resistance can be primary, as in never-responders, or acquired, which emerges after a period of response. Resistance can also be classified as intrinsic or extrinsic to tumor cells. Intrinsic resistance is seen when cancer cells alter processes that are related to immune recognition, cell signaling, gene expression, and DNA damage response. Extrinsic resistance occurs external to tumor cells throughout the T-cell activation process. Cancer immunotherapy blocking PD-1/PDL-1 check point may be ineffective and this effect may be attributable to the development of resistance mechanism involving different cells of the tumor microenvironment, including mast cells.

Bioinformatics analysis of patients with melanoma was used to evaluate the prognostic value of mast cells and their association with anti-PD-1 immunotherapy (Li et al. 2022). Results confirmed that mast cells may be considered as a poor prognostic factor and are associated with resistance to anti-PD-1 immunotherapy. Activation of mast cells induced by anti-PD-1 antibody was partially inhibited by cromolyn sodium. In a mouse subcutaneous melanoma model, cromolyn sodium inhibited the activation of mast cells treated with PD-1 antibody and increased the efficacy of immunotherapy.

Mast cells may be responsible in a humanized-mouse melanoma model for resistance to anti-PD-1 therapy, which is linked to lower expression of HLA class I, resulting in tumor escape from cytotoxic T cells (Somasundaram et al. 2021). Immunostaining of human melanoma patients’ tumors showed an increased number of mast cells after anti-PD1 therapy, when compared to untreated individuals. Examination of tumor tissue sections after anti-PD1 therapy demonstrated a co-localization of mast cells and forkhead box P3 (FOXP3)^+^ Treg cells and this evidence may be associated with a down-modulation of HLA class I on tumor cells. Reduced HLA class I expression and CD8^+^/Granz B^+^ T cells homeostasis are observed in regions where FOXP3^+^ Treg cells and mast cells co-localize and are associated with resistance to anti-PD1 treatment. Melanoma cells secrete chemokine (C-X-C motif) ligand 10 (CXCL10) that bind CXC motif chemokine receptor 3 (CXCR3), a chemokine receptor expressed by mast cells, favoring the recruitment of mast cells to the tumor site (Kuo et al. 2018).

## Mast Cells and Resistance to Immunotherapy in Combination with Radiotherapy and Chemotherapy

The use of T-cell stimulatory immunotherapies or small-molecule inhibitors of immunosuppressive signaling pathways in the tumor microenvironment in combination with radiotherapy and/or chemotherapy may be a useful strategy in the treatment of cancer patients in the long term.

Prostate cancer recruits more mast cells than normal prostate epithelial cells, leading prostate cancer more resistant to chemotherapy and radiotherapy. Mast cells can induce docetaxel resistance in prostate cancer by phosphorylating p38 and radio-resistance by phosphorylating ataxia-telangiectasia mutated kinase (a master controller of signal transduction) resulting in tumor cell survival and proliferation (Xie et al. 2016). Combining anti-PD-1 with sunitinib or imatinib, both inhibitors of receptor tyrosine including cKIT  expressed by mast cells, resulted in the depletion of mast cells and complete regression of tumor.

Cancer-associated fibroblasts and TGF-β signaling activation by mast cells contribute to resistance to gemcitabine/nabpaclitaxel in pancreatic cancer (Porcelli et al. 2019). Poor response to neo-adjuvant chemotherapy correlates with mast cell infiltration in inflammatory breast cancer (Reddy et al. 2019). Tryptase-positive mast cells were significantly higher in non-responders than responders’ patients. Mast cells may exert their inhibitory effect through suppressing CD8^+^ cells, enhancing immunosuppressive CD163^+^ macrophages, and directly promoting tumor cell growth.

Mast cells decrease efficacy of anti-angiogenic therapy by secreting matrix degrading granzyme B (GZMB), which liberates pro-angiogenic factors, such as FGF-1 and granulocyte–macrophage colony stimulating factor (GM-CSF) from the extracellular matrix. GM-CSF, in turn, exert chemoattraction for neutrophils, monocytes, and lymphocytes, which release other angiogenic factors (Wroblewski et al. 2017). Treatment with DC101, an anti-VEGF receptor-2, reduced tumor volume in the absence of mast cells. Anti-angiogenic therapy did not change the number or degranulation status of tumor-infiltrating mast cells. DC101 treatment increased GZMB and decreased tumor volume more pronouncedly when mast cells are deficient for GZMB. An additive inhibitory effect of DC101 on tumor growth has been obtained when cromolyn, an inhibitor of mast cell degranulation was  added.

## Mast Cells, Myeloid-Derived Suppressor Cells and Immunosuppression

Mast cells can contribute to immunosuppression of solid tumor sites, through mediator’s release and through their interactions with Treg and myeloid-derived suppressor cells (MDSCs). Crosstalk between MDSCs and mast cells mediates tumor-specific immunosuppression in prostate cancer (Jachetti et al. 2018). CD40L expressing mast cell interaction with MDSCs lead to T-cell suppression. In fact, mast cells can augment MDSC suppressive functions, and these cells can interact by binding of CD40L and CD40 (Danelli et al. 2015). These data suggest that CD40L–CD40 signaling may sustain immunosuppression in those cancer patients characterized by accumulation of both mast cells and MDSCs. In this context, CD40L–CD40 signaling may sustain immunosuppression and blocking CD40L–CD40 interaction with antagonistic CD40 antibodies, such as Lucatumumab, may increase the anti-tumor immune response and may be tested in combination with immunotherapy (Byrd et al. 2012).

Mast cells recruit MDSCs and induce their production of IL-17, which in turn acts to mobilize Treg and enhances their suppressor function and induced IL-9 production that, in turn, stimulates the survival and pro-tumoral effects of mast cells. Moreover, MDSCs release MMP-9 through which SCF is generated, that further recruit mast cells in the tumor site (Yang et al. 2010).

## Concluding Remarks

Very little is currently known about the phenotype and function of tumor-infiltrating mast cells. Mast cells are among the first immune cells recruited to solid tumor sites, are increased in pre-cancerous lesions, and found in greater abundance as cancer progresses (Ribatti and Crivellato 2009).

Abnormal signals from the tumor microenvironment favor the initiation and promotion of neoplastic transformation. Once tumor is formed, it modifies the stroma and initiates an inflammatory reaction and an immune response. In this context, under a variety of conditions, mast cells are capable of polarize them under their tumoricidal (growth arresting) or tumorigenic (growth promoting) forms. The establishment of immunosuppressive pathways in the tumor microenvironment has pointed therapeutic strategies that may promote inflammation and/or innate immune activation in the tumor microenvironment.

Mast cells are ideal candidates for targeted tumor immunotherapy due to their abundance at in many solid tumors, their proximity to blood vessels and capacity to selectively secrete distinct profiles of mediators. Mast cells can be therapeutically targeted by decreasing cell numbers through c-KIT inhibition, modulating mast cell activation and phenotype (through mast cell stabilizers, FcεR1 signaling pathway activators/inhibitors, antibodies targeting inhibitory receptors and ligands, tool-like receptor (TLR) agonists), and altering secreted mast cell mediators and their downstream effects.

Otherwise, mast cells can modulate anti-tumor immunity through the release of granule-associated and de novo synthesized mediators that induce the mobilization of immune cells and their activation and differentiation. In this context, mast cells can contribute to the immune suppressive tumor microenvironment via mobilization of and interaction with MDSC and Treg. Mast cells may also enhance anti-tumor responses via the recruitment of natural killer (NK) cells, dendritic cells (DCs), and T cells and via their interactions with these cells to enhance their activation. More recently, it has been demonstrated that mast cells can infiltrate solid tumors and kill cancer cells within tumors, confirming their role in cancer immunotherapy (Fereydouni et al. 2022a, b).

**Fig. 3:**
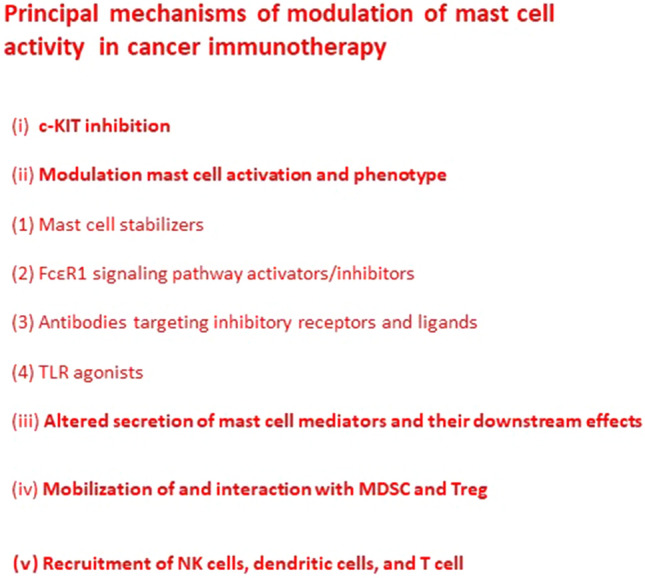
Principal mechanisms of modulation of mast cell activity in cancer immunotherapy

For these reasons, it is extremely important to know the mechanisms that are involved in the development and involvement of mast cells in the resistance to conventional immunotherapies (Fig. 3). This concerns will allow to better analyze and suggest alternative cancer therapeutic strategies in which mast cells may be considered as an active player together with other cells in the tumor inflammatory microenvironment.
